# The Sesquiterpenes β-Caryophyllene and Caryophyllene Oxide Isolated from *Senecio salignus* Act as Phytogrowth and Photosynthesis Inhibitors

**DOI:** 10.3390/molecules17021437

**Published:** 2012-02-06

**Authors:** B. Arturo Sánchez-Muñoz, Maria Isabel Aguilar, Beatriz King-Díaz, José Fausto Rivero, Blas Lotina-Hennsen

**Affiliations:** 1 Departamento de Bioquímica, Facultad de Química, Universidad Nacional Autónoma de México, Ciudad Universitaria, Delegación Coyoacan, México D. F. 04510, Mexico; 2 Departamento de Farmacia, Facultad de Química, Universidad Nacional Autónoma de México, Ciudad Universitaria, Delegación Coyoacan, México D. F. 04510, Mexico

**Keywords:** *Senecio salignus*, *β*-caryophyllene, caryophyllene oxide, phytogrowth inhibitor, photosynthesis, Chlorophyll *a* fluorescence

## Abstract

The *n*-hexane extract of *S. salignus* plants inhibited ATP synthesis and two sesquiterpenes, the β-caryophyllene (**1**) and caryophyllene oxide (**2**) were isolated from this nonpolar fraction. Compound **1** inhibited by 42% the root elongation of *Physalis ixocarpa* seedlings at 50 µg/mL and by 53% at 150 µg/mL, whereas at 150 µg/mL this compound only inhibited root elongation of *Echinochloa crus-galli* by 30%. On the other hand, compound **2** had no effect on either germination or root and stem growth of *E. cruss galli* and *P. ixocarpa*. However, **1** and **2** inhibited the dry biomass of *P. ixocarpa* plants grown for 18 days previous to treatment and it was found that **1** was the most active biomass inhibitor. The Chl *a* fluorescence transient *in vivo* experiment indicates that **1** (100 µg/mL) has a major effect at 72 h after treatment on leaves of *P. ixocarpa* plants by inhibiting photosystem II (PS II) transforming active reaction centers to “heat sinks” or the formation of silent reaction centers unable to reduce Q_A_. β-Caryophyllene also induces chlorosis on treated leaves.

## 1. Introduction

The genus *Senecio* includes approximately 1,500 species widely distributed in Mexico and Central America [1]. Among the many species, the synonymous genus *Barkleyanthus* only has a single species: *B. salicifolius or Senecio salignus* (H.B.K.) Rob. and Brett. Quercetin was isolated from *S*. *salignus* (*B. salicifolius*) [2]. Furoeremophilane epoxides were also isolated from the aerial parts of *S. salignus* and 7-ketofuranoeremophilane from the roots in 1976 [3]. Later, the same authors found the pyrrolizidine alkaloid 7-angelylheliotridine [4]. *S. salignus* leaves are used against intermittent fever and rheumatism [5]. Our search for secondary metabolites in plants that affect photosynthesis, identified two sesquiterpenes: β-caryophyllene (**1**) and caryophyllene oxide (**2**) in *S. salignus*. It is well known that β-caryophyllene has anti-inflammatory, insecticidal and fungicidal [6] activities. In this work, compound **1** inhibited the germination and growth of *Physalis ixocarpa*, as well as the growth (root and stem elongation) of *Echinocloa crus-galli*. *In vitro* assays showed that sesquiterpene **1** also inhibited photosynthetic activities; however, compound **2** only inhibited *Physalis ixocarpa* plant growth, root and stem elongation. Light reactions of photosynthesis were performed by fluorescence induction curves of chlorophyll *a* of photosystem II (PS II), as shown by a JIP test.

## 2. Results and Discussion

### 2.1. Sesquiterpene Isolation and ATP Synthesis Determination

**Figure 1:**
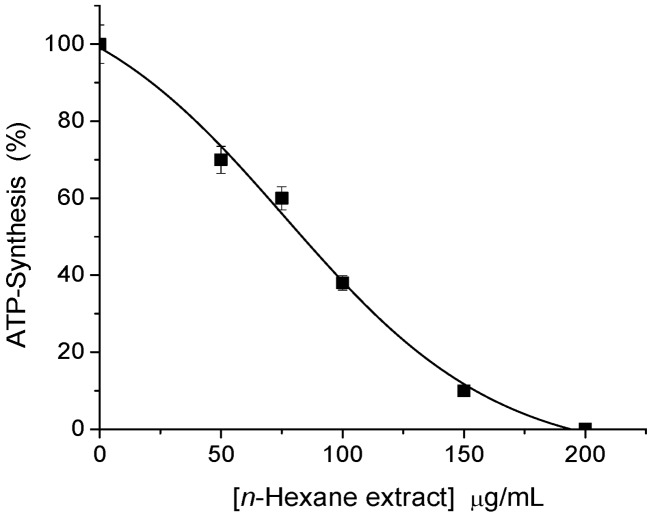
Inhibitory effect of increasing concentrations of *n*-hexane extract (obtained from aerial parts of *S. salignus*) on ATP synthesis rate of spinach thylakoids. Control value was 971 µM ATP / mg Chl × h. The data are results of three replicates.

In the screening bioassay (ATP synthesis) it was found that the *n*-hexane extract (leaves and stems) of *S. salignus*, exhibited the major inhibition result (I_50_ 79.3 µg/mL) (Figure 1), indicating that it might contain secondary metabolites with inhibitory activity on photosynthesis.

**Figure 2:**
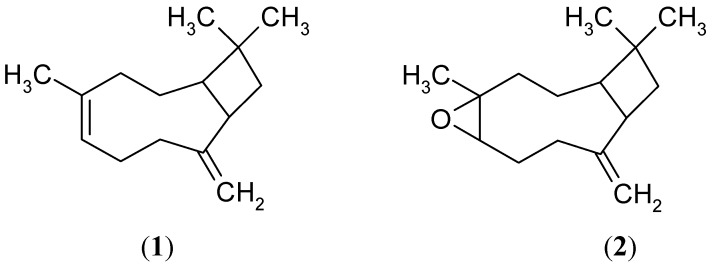
Structures of β-caryophyllene (**1**), and caryophyllene oxide (**2**).

F30-F54 of the primary fraction obtained from a chromatographic column inhibited ATP synthesis with an I_50_ value equal to that obtained for the extract. In order to know the compounds involved in this active fraction, a secondary chromatographic fractionation eluting with an ascendant gradient of *n*-hexane–EtOAc 80:20, and then with acetone 100%, gave a mixture of compounds, spectroscopically identified as β-caryophyllene (**1**, 3 mg) and caryophyllene oxide (**2**, 2 mg) [7] (Figure 2).

### 2.2. Effects of **1** and **2** on Germination and Root and Stem Growth of *E. cruss-galli* and *P. ixocarpa*

As concentration of caryophyllene oxide increased root elongation of *E. crus-galli* was partially inhibited (23 and 30% at 100 and 150 µg/mL respectively); the significance values were 0.014 and 1.21 × 10^−4^ (obtained from the statistical *t* test for two populations method). The root growth of *P. ixocarpa* plants was inhibited by 42 to 53%, when concentrations of **1** were varied from 50 to 150 µg/mL with *p* values of 0.0046 and 1.94 × 10^−4^ (Table 1). These results are in agreement with the previously reported inhibition of seed germination, root and shoot growth of *Brassica. campestris*, *Raphanus sativus*, *Lactuca sativa*, *Mikania micrantha* and *Achyranthes japonica* [8]. On the other hand, compound **2** had no effect on either germination and root and stem growth of *E. cruss-galli* and *P. ixocarpa*.

**Table 1:** β-Caryophyllene inhibition of root, shoot and germination of *P. ixocarpa* seedlings.

Conc. [µg/mL]	Root Elongation (%) *p*		Shoot Elongation (%) *p*		Germination (%)
50	42	0.0046	16	0.075	0
100	44	6.04 × 10^−4^	18	0.06	0
150	53	1.94 × 10^−4^	19	0.064	4

### 2.3. Postemergence Activity of **1** and **2** on Biomass Production of *P. ixocarpa* and *L. perenne* Plants

**Figure 3:**
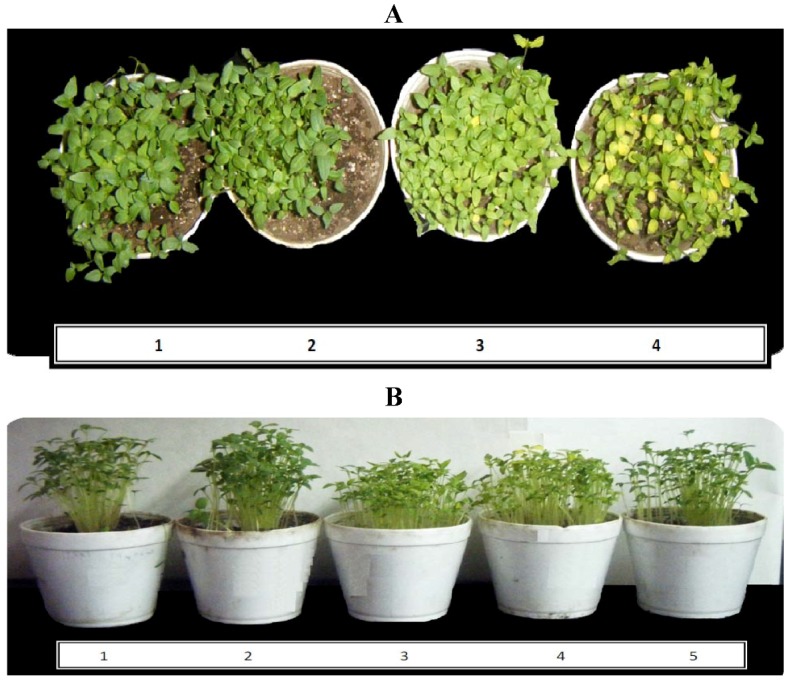
(**A**) Bleaching of the *P. ixocarpa* leaves observed on plants after one week treated with: (1) Control; (2) 50 µM DCMU; (3) 50 and (4) 100 µg/mL of β-caryophyllene. (**B**) shows the plants growth under the effect of: (1) control; (2) control with the solvent (EtOH); (3) 50 and (4) 100 µg/mL of β-caryophyllene and (5) 50 µM DCMU.

Compounds **1** and **2** acted inhibiting mainly the dry biomass production of *P. ixocarpa* plants (Table 2). Compound **1** (100 µM) inhibited the dry biomass of *P. ixocarpa* plants by 37%, and **2** only by 22%, suggesting that the double bond between carbons 4–5 in the β-caryophyllene molecule is necessary for activity. *L. perenne* plants were less inhibited by both sesquiterpenes. Figure 3A shows bleached leaves of *P. ixocarpa* plants treated with **1**, after one week and the elongation of the plant decreased (Figure 3B), which are in agreement with the dry biomass and bleaching effect results reported for monoterpene derivatives of another Asteraceae species plant family [9] which inhibits phytoene desaturase, a key enzyme in carotenoid pigment biosynthesis. Since carotenoids protect chlorophyll from photooxidation, their lack would result in loss of chlorophyll. The bleaching of *P. ixocarpa* leaves caused by β-caryophyllene suggests that it could act as a carotenoid biosynthesis inhibitor. *L. perenne* plants were less inhibited (15% and 4%) with **1** and **2** (100 µg/mL).

**Table 2:** Effects of **1** and **2** on biomass production *in vivo* (measured as dry weight of *P. ixocarpa* and *L. perenne* plants grown previously for 18 days before treatment). The data are results of five replicates.

	*P. ixocarpa*		*L. perenne*	
Comp / Conc.	G	%	g	%
Control	0.7203 ± 0.184	100	1.068 ± 0.031	100
DCMU	0.533 ± 0.053	74	0.897 ± 0.054	84
(**1**) / [µg/mL]				
25	0.663 ± 0.03	92	1.107 ± 0.142	104
50	0.575 ± 0.021	80	0.879 ± 0.132	82
100	0.45 ± 0.104	63	0.908 ± 0.197	85
(**2**) / [µg/mL]				
25	0.685 ± 0.055	95	0.953 ± 0.111	89
50	0.654 ± 0.065	91	0.962 ± 0.117	90
100	0.657 ± 0.02	78	1.02 ± 0.111	96

### 2.4. Localization of the Target of 1 on Photosynthetic Electron Transport Chain Studied by the Fluorescence of Chl a *in Vivo* Technique on Leaves of *P. ixocarpa* and *L. perenne* Plants

**Figure 4:**
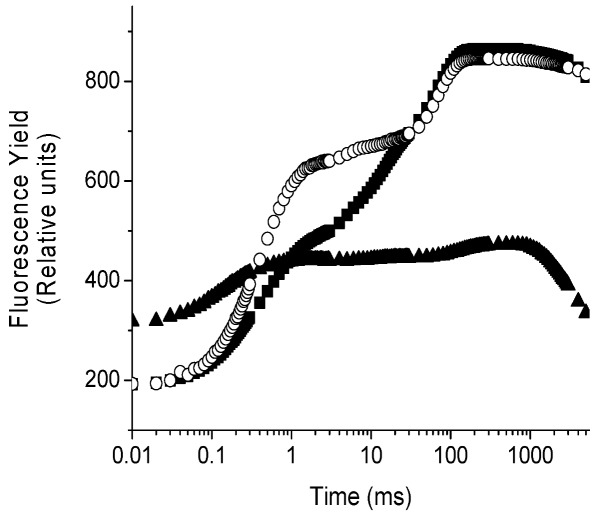
Fluorescence rise kinetic curves of *P. ixocarpa* leaves measured 72 h after treatment with **1**, 100 µg/mL (▲), control (■) and 50 µM DCMU (○). Data are an average of ten replicates.

Chlorophyll *a* fluorescence is an intrinsic evidence of the photosynthetic system and the intensity of the fluorescence is a direct measure of the PS II activity. The fluorescence induction curve of intact leaves of *P. ixocarpa* and *L. perenne* plants, exhibited a polyphasic rise O-J-I-P transient (control leaves) (Figure 4) [10]. The addition of the herbicide DCMU, used as positive control, resulted in a fast rise of fluorescence yield during the first 2 ms of illumination, transforming the regular O-J-I-P sequence into an O-J curve (Figure 4) [10]. The plot on a logarithmic time scale revealed that there are large differences between the control and samples treated with 100 μM of **1**, 72 h after treatments (Figure 4).

**Figure 5:**
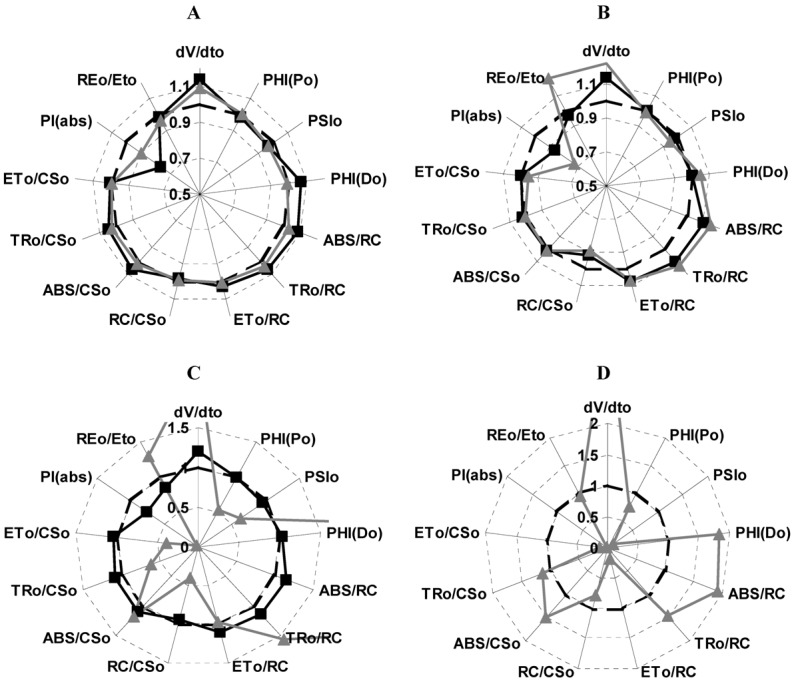
Effect of **1** at 50 (■) and 100 µg/mL (▲) on different calculated parameters from OJIP curves measured on *P. ixocarapa* plants treated after 24 h (Panel A), 48 h (Panel B) and 72 h (Panel C), and 50 µM DCMU (Panel D) after 72 h of treatment.

From the kinetics data of the fluorescence curve measured at 24, 48 and 72 h after treatment on leaves and compared with the control, various parameters were calculated and plotted as a radar graph, a circular graphic with a series of spokes or rays projecting from a central point, with each ray label representing a different variable (Figure 5). Data values were normalized to a common specific range or percentile values. Figure 5 shows the parameters (Strasser *et al.* [11]) of *P. ixocarpa* plants affected with **1**, Panel A shows the effects at 24 h of treatment. Performance index decreased 25% and 10% at 50 and 100 µg/mL, respectively; and the absorption per reaction center (ABS/RC), the quantum yield for energy dissipation PHI(D_0_), the trapped energy flux per reaction center TR_0_/RC and the indicator of the water splitting enzyme function (dV/dt_0_) all were increased 10% at 100 µg/mL. Furthermore, at 72 h of treatments of *P. ixocarpa* plants with **1** (100 µg/mL): The electron transport per cross section (ET_0_/CS) and the maximum quantum yield of primary photochemistry [PHI(P_0_)] decreased 50% (Figure 5, Panel **C**). The probability that a trapped exciton moves an electron into the electron transport chain beyond Q_A_− (PSI_0_), the density of the reaction centers (RC/CS_0_), the trapping and the electron transport per cross section (TR_0_/CS and ET_0_/CS) decreased around 50%; while the following parameters increased up to 20%: dV/dt_0_, ABS/RC and TR_0_/RC. These observations suggested that some PSII RCs were transformed to “heat sinks” or “silent reaction centers”; these centers can neither reduce Q_A_ and their excitation energy is dissipated as heat [11], this is the reason why PHI(D_0_) values increase. The mechanism of action of **1** is similar to other natural products like robustaflavone [12]. In panel D the effects of 50 µM of DCMU on the parameters are shown to compare them.

Table 3, shows how the active reaction centers (RC^Si^), calculated as [(ABS/RC)_C_/(ABS/RC)_T_ × 100] for each treatment; [(ABS/RC)_C_ means for control value and the (ABS/RC)_T_ means the treatments values] decreased in function of time of treatment. Thus **1** (100 µM) at 72 h of treatment decreased 86%, and the increased value of the RE/ET_0_ parameter is due to the lack of the reduced equivalents of PS II (Figure 5, Panels B and C).

**Table 3:** Active Reaction Centers calculated as [(ABS/RC)_C_ /ABS/(RC)_T_] × 100 in control and treated *P. ixocarpa* plants with β-caryophyllene 50 and 100 mg/mL.

	24 h		48 h		72 h	
(1) [µg/mL]	ABS/RC	Active RC (%)	ABS/RC	Active RC (%)	ABS/RC	Active RC (%)
0	1.91 ± 0.19	100	2.053 ± 0.25	100	2.282 ± 0.27	100
25	1.736 ± 0.11	110	1.992 ± 0.14	103	1.888 ± 0.08	120
50	2.09 ± 0.22	91	2.273 ± 0.13	90	2.66 ± 0.5	86
100	1.98 ± .25	96	3.323 ± 1.49	62	16.13 ± 3.4	14

## 3. Experimental

### 3.1. General

#### 3.1.1. Reagents

Reagents were purchased from Sigma-Aldrich and the salts from J. T. Baker.

#### 3.1.2. Methods

##### 3.1.2.1. Tested Material

Aerial parts of *S. salignus* were collected in the state of Guerrero, Mexico in 2004 (R. Santiago, collection No R1995) and a voucher specimen was deposited at *Facultad de Ciencias UNAM*. No 115634. The aerial parts of *S. salignus* were dried, powdered and extracted exhaustively by maceration at room temperature with *n*-hexane. The resultant crude extract (22 g) inhibited ATP synthesis (I_50_ = 79.3 µg/mL) (Figure 1) and was fractionated via column chromatography on silica gel (0.0063–0.200 mm), and eluted with a mixture of increasing polarity gradient of *n*-hexane–EtOAc. The fractions eluted with *n*-hexane–EtOAc 90:10 were subjected to a second fractionation and eluted with mixtures of *n*-hexane–EtOAc of ascendant polarity, and β-caryophyllene and caryophyllene oxide were isolated with hexane–EtOAc 80:20 (Figure 2). The compounds structures were identified by spectroscopic methods with a Bruker DRX 400spectrometer for NMR, for mass spectra an Shimadzu QP5050A device for electron impact (70 eV) low-resolution (EI-MS) was used and for gas chromatography/mass (GC/MS) spectrometry an Shimadzu QP-5050A, equipped with a PTE™-5 was used, to give values consistent with data reported in the literature [7].

##### 3.1.2.2. Chloroplast Isolation and Chlorophyll Determination

Intact chloroplasts were isolated from market spinach leaves (*Spinacea oleracea*) as reported [13,14]. Chloroplasts were suspended in a small volume of the following solution: 400 mM sucrose, 5 mM MgCl_2_, 10 mM KCl, and 30 mM *N*-Tris[hydroxymethyl)methyl]glycine (tricine) pH 8.0 with KOH addition. Chloroplasts were stored as concentrated suspension in the dark for 1 h at 4 °C. The chlorophyll (Chl) concentration was measured according to Strain *et al.* [15].

##### 3.1.2.3. Determination of ATP Synthesis

Intact chloroplasts (20 μg of Chl /mL) were broken before each assay by osmotic rupture in a solution (3 mL) containing: 100 mM sorbitol, 10 mM KCl, 5 mM MgCl_2_, 0.5 mM KCN, 1 mM tricine-KOH at pH 8.0 in the presence of 50 μM methylviologen (MV) and 1 mM adenosine diphosphate (ADP) at pH 6.5. The pH was adjusted to 8.0 with 50 mM KOH and ATP synthesis was titrimetrically determined using a microelectrode (Orion Mod. 8103 Ross) connected to a potentiometer (Corning Medical, model 12, Acton, MA, USA) with an expanded scale and a Gilson recorder (Kipp & Zonen, Bohemia, NY, USA). Alkalization rates were measured in the linear part during illumination. The reaction was calibrated by back titration with saturated HCl. The reaction started when turning the light on in the presence of chloroplasts (20 µg of chlorophyll per mL) [14,16]. The I_50_ value (concentration producing 50% inhibition) for each activity was determined from plots of the activity at different concentrations of compound.

##### 3.1.2.4. Chlorophyll a Fluorescence of PS II

Chlorophyll fluorescence induction curves were measured at room temperature with a Hansatech Handy PEA (Plant Efficient Analyzer) as previously described [12]. The maximum fluorescence yield of PS II was generated using three light-emitting diodes (broad 650 nm). The pulse duration was 2 s, with intensity of 2830 nm and gain of 0.7. Chlorophyll fluorescence determination *in vivo*, in intact and dark adapted (30 min) leaves, was performed for control and sprayed plants with concentrations of 25, 50 and 100 µg/mL of **1** and **2**, after 24, 48 an 72 h of treatments. Data were analyzed and processed with Handy PEA and Biolyzer programs to obtain different photosynthetic parameters associated to PSII, according to the O-J-I-P test equations [14,17]: F_0_ is the Fluorescence intensity level at 20 μs when plastoquinone electron acceptor pool (Q_A_) is fully oxidized; Fm is the Fluorescence level when Q_A_ is transiently fully reduced; M_0_ is an indication of the water splitting enzyme function calculated as dV/dt_0_ = 4[(F_300_ − F_0_)/(F_M_ − F_0_)]; ABS/RC is the absorption of photons flux (ABS) per active reaction center (RC) showing the antenna size equal to (M_0_/V_J_)/PHI(P_0_); ET/RC is the electron transport rate per active reaction center calculated as (M_0_/V_J_)(1 − V_J_); TR/RC is the trapped energy flux per RC (at t = 0) equal to M_0_(1/V_J_); dR/RC is the efficiency when an electron moves from the reduced PSII intersystem electron acceptors to the PSI electron acceptors end calculated as (1 − V_I_/1 − V_J_); RE/RC is the reduction of the PSI electron acceptor end side per RC at t = 0 calculated as (dR/RC)(ET/RC); PHI(P_0_) is the maximum quantum yield of primary photochemistry at P transient (t = 0), it measures the PS II efficiency at plastoquinone pool reduction equal to 1 − (F_0_/F_M_); PSI_0_, calculated as 1 − V_J_ is the probability (at t = 0) that a trapped exciton moves an electron into the electron transport chain beyond Q_A_^−^; PHI(D_0_) is the quantum yield (at t = 0) of energy dissipation determined as (F_J_ − F_0_)/(F_M_ − F_0_); V_J_, is the fluorescence quantum yield at J transient and used as an indication of PS II efficiency in the primary photochemistry; V_I_ is the relative variable fluorescence at 30 ms calculated as (F_30 ms_ − F_0_)/(F_M_ − F_0_); RC/CS determined as PHI(P_0_)(V_J_/M_0_)(ABS/CS), is the density of the reaction centers; ABS/CS equal to F_0_, a phenomenological energy flux is the absorption flux per excited cross section; RC/ABS is the fraction of reaction center Chl molecules relative to the total Chl content; PI(abs) is the performance index (PI) on absorption basis calculated as [RC/ABS] × [PHI(P_0_)/(1 − PHI(P_0_)] × [PSI_0_/(1 − PSI_0_)].

##### 3.1.2.5. Seed Germination Bioassays

Monocot seeds of *Echinochloa crus galli* L. P. Beauv. an annual grass and dicotyledon seeds of *Physalis ixocarpa* L. (green tomato) were purchased from Semillas Berentsen, S. A. de C. V. (Celaya, Guanajuato, Mexico). Germination tests were run in triplicate with 40 *E. crus galli* seeds and 40 *P. ixocarpa* seeds for each concentration of sample for five days (three days for germination and two days more for root and shoot growth). The number of seeds used for each experiment was selected for that show an appreciable change in O_2_ uptake that could be detected by the oxygraph (Yellow Spring Instrument) Model 5300. The test seeds were held in the dark at 28 °C in 9.0-cm Petri dishes containing an 8.5-cm sheet of Whatman No. 1 filter paper and 10.0 mL of test or control solution. Dishes were wrapped with Parafilm foil and incubated at 28 °C in the dark. The number of germinated seeds was determined according to the criterion of 1 mm extrusion of the radicle. Germination rates were counted at 72 and 120 h later for root and shoot growth measurements. **1** and **2** were initially dissolved in dimethyl sulfoxide (DMSO). The maximum final concentration of DMSO was less than 0.1% and the same DMSO concentration was used in the control solution [18].

##### 3.1.2.6. Plant Material for *in Vivo* Assays

The seeds of *P. ixocarpa* and *L. perenne*, were sown in 12 cm diameter pots and were watered daily in the greenhouse at 27 ± 2 °C. After 15 and 18 days of emergence for *P. ixocarpa* and *L. perenne*, respectively, the plants were selected for similar size and were sprayed manually with the **1** and **2** at concentrations of 25, 50 and 100 µg/mL (one stock of 10 mg/mL of each compound was prepared in DMSO). An aliquot of the stock solution was taken to obtain the desired concentration in an aqueous suspension containing 0.05% w/v of polyoxyethylenesorbitan monolaurate (Tween-20). The control samples were sprayed with distilled water containing the same amount of DMSO and Tween-20 [19].

## 4. Conclusions

Allelochemicals play a role in mediating interspecific interactions [20,21], and various plants enhance competitiveness and fitness [22,23]. β-caryophyllene (**1**), a well-known volatile sesquiterpene with allelopathic potential, has been reported to inhibit development of seedlings of various plant species [24,25]. In this work, for the first time from the *n*-hexane extract of aerial parts of *S. salignus* plants, were isolated β-caryophyllene and caryophyllene oxide. Compound **1** inhibited 42% the root elongation of *Physalis ixocarpa* seedling at 50 µg/mL and 53% at 150 µg/mL and it inhibited the root elongation of *Echinochloa crus-galli* to a lesser extent (30% at 150 µg/mL). On the other hand, compound **2** was inactive in both seedlings. Both compounds inhibited the dry biomass of *Physalis ixocarpa* plants, being β-caryophyllene the most active. The Chl *a* fluorescence transient *in vivo* indicated that 100 µg/mL of β-caryophyllene presented a major effect on photosynthesis of *P. ixocarpa* plants 72 h after treatment, inhibiting PS II by transforming active reaction centers to “heat sinks” or the formation of silent reaction centers, and also induces chlorosis on treated leaves.
